# Are there really no causal associations between childhood family income and subsequent outcomes?

**DOI:** 10.1093/ije/dyac016

**Published:** 2022-02-13

**Authors:** Anders Ledberg, Kristiina Rajaleid, Bitte Modin

**Affiliations:** Department of Public Health Sciences, Centre for Health Equity Studies (CHESS), Stockholm University, Stockholm, Sweden; Department of Public Health Sciences, Centre for Health Equity Studies (CHESS), Stockholm University, Stockholm, Sweden; Department of Psychology, Stress Research Institute, Stockholm University, Stockholm, Sweden; Department of Public Health Sciences, Centre for Health Equity Studies (CHESS), Stockholm University, Stockholm, Sweden

We read with interest the recent paper by Sariaslan *et al*.^1^ investigating the associations between childhood family income and three outcomes observed later in life: psychiatric disorders, substance misuse and violent crime arrests. The paper contains a careful analysis of a large and relevant dataset of more than 650* *000 individuals born in Finland in 1986–96, who were followed from age 15, when income was assessed, until 2017 or 2018. The authors use two types of analysis in the paper: one in which differences in the outcomes are accounted for by variability in income between families (henceforth between-family analysis) and one, the so-called sibling comparison, in which outcome differences are accounted for only by income variability within families (henceforth within-family analysis). In the between-family analysis, the authors find consistent associations between a number of related estimates of childhood family income and the three outcomes (e.g. Figure 2 in the paper). However, and this is the main finding of the paper, these associations between income and the three outcomes completely disappear in the within-family analysis. From this, the authors conclude that ‘[a]ssociations between childhood family income and subsequent risks … were not consistent with a causal interpretation’.

We have no major objections to the results, but we believe that the authors are mistaken about what can be inferred from the null results of the within-family analysis. In fact, it is well established that between- and within-family analyses might, in general, be sensitive to different types of causal effects.^2^ The authors’ conclusions, therefore, do not follow from their results. In the jargon of causal inference, a within-family analysis conditions both on family-level confounders (which is desired) and mediators, i.e. variables through which a causal effect of income might be channelled.^2^ This implies that the within-family analysis tests for direct effects^3^ of income, i.e. causal effects that are not mediated by conditions common to siblings within the same family. The between-family analysis, on the other hand, is sensitive to the total effect of income (but also to confounders). The authors could therefore arguably conclude that there is no evidence in their data for a direct causal effect of childhood family income on the three outcomes (although, see Rod *et al*.^4^). In general, however, their results are also fully consistent with there being a total effect of childhood family income on each of the three outcomes.

To illustrate our point, assume that childhood family income has a causal effect on the neighbourhood in which a family resides while the children are growing up: the higher the income, the more affluent the neighbourhood. Assume further that neighbourhood has a causal effect on one of the outcomes studied by the authors, say criminal behaviour: the poorer the neighbourhood, the higher the risk of developing criminal behaviour. The first assumption is uncontroversial; the second may or may not be true, here we are just assuming that it is. Next, consider two families: the high-income family R, with children Ron and Ruth, living in a rich neighbourhood, and the low-income family P, with children Peter and Paula, living in a poor neighbourhood. Assume that the annual family income the year when these children turned 15 was as follows (in units of 1000 USD): Ron: 120; Ruth: 126; Peter 34; Paula 40. Note that, in a between-family analysis, the large difference in the average income between these families (123 versus 37) also conveys information about the neighbourhood in which these families reside. By assumption, we would therefore expect to find an association between income and criminal behaviour in this analysis. In a within-family analysis, on the other hand, it is only deviations from the average family income that matter for the analysis (Ron: –3; Ruth: 3; Peter: –3; Paula: 3). Consequently, the income data no longer carry any information about neighbourhoods, and the within-family analysis is therefore blind to the assumed causal effects of income mediated by this variable.

In conclusion, the paper by Sariaslan *et al*. provides a valuable contribution to the understanding of the pathways through which childhood family income exerts its potential effects on future outcomes. However, the null findings of the within-family analysis do not support the far-reaching conclusions conveyed by the authors.

## Author contributions

A.L, K.R. and B.M read and discussed the paper^1^ and agreed to write a letter to the Editor in response. A.L. and B.M drafted the first version, and K.R. helped revising the draft. All authors agreed on the final version.

## Funding

This work was supported by the Swedish Research Council for Health, Working Life and Welfare (Grant No. 2016–07148).

## Conflicts of interest

None declared.

## References

[1] Sariaslan A , Mikkonen J, Aaltonen M, Hiilamo H, Martikainen P, Fazel S. No causal associations between childhood family income and subsequent psychiatric disorders, substance misuse and violent crime arrests: a nationwide Finnish study of >650 000 individuals and their siblings. Int J Epidemiol 2021;50:1628–38. 34050646 10.1093/ije/dyab099 PMC8580272

[2] Sjölander A , Frisell T, Öberg S. Sibling comparison studies. Annu Rev Stat Appl 2022;9: 10.1146/annurev-statistics-040120-024521. PMC9209381 35312926

[3] Pearl J. Direct and indirect effects. In: *Proceedings of the Seventeenth Conference on Uncertainty in Artificial Intelligence*, *UAI’01, 2*–5 *August 2001.* Seattle, WA. San Francisco, CA: Morgan Kaufmann, 2001, pp 411–20.

[4] Rod NH , Lange T, Petersen AH. Do sibling comparisons answer the causal question? In response to ‘No causal associations between childhood family income and subsequent psychiatric disorders, substance misuse and violent crime arrests’. Int J Epidemiol 2022;51:2025–26. 10.1093/ije/dyab235 34751751

